# Menstrual health challenges and associated barriers among tribal adolescent girls in India: A systematic review

**DOI:** 10.1371/journal.pone.0360401

**Published:** 2026-10-12

**Authors:** Rohit Raj, Jarina Begum, Syed Irfan Ali, Manasee Panda, Vani Lakshmi R

**Affiliations:** 1 Department of Community Medicine, Manipal Tata Medical College, Manipal Academy of Higher Education, Manipal, India; 2 Odisha University of Health Sciences, Bhubaneswar, India; 3 Centre for Digital Health, Applied Research and Technology, Prasanna School of Public Health, Manipal Academy of Higher Education, Manipal, India; Cranfield University, UNITED KINGDOM OF GREAT BRITAIN AND NORTHERN IRELAND

## Abstract

**Introduction:**

Menstrual health is a critical yet neglected public health issue among tribal adolescent girls in India, influenced by socio-cultural, economic, and infrastructural determinants. This review aimed to explore the key menstrual health challenges, barriers, and associated outcomes among tribal adolescent girls in India.

**Methods:**

This systematic review was conducted in accordance with PRISMA 2020 guidelines. A comprehensive literature search was performed across PubMed, Scopus, and Google Scholar for studies published between January 2015 and March 2026. Quantitative, qualitative, and mixed-methods studies focusing on menstrual health among tribal adolescent girls in India were included. Study selection, data extraction, and quality appraisal were conducted independently by multiple reviewers. Methodological quality was assessed using the Joanna Briggs Institute (JBI) tools. Due to heterogeneity, a narrative synthesis approach was adopted.

**Results:**

Out of 197 identified records, 28 studies met the inclusion criteria. The findings reveal significant disparities in menstrual health management driven by socioeconomic status, education, cultural taboos, and infrastructural limitations. Key challenges included limited access to sanitary products, inadequate WASH facilities, and poor awareness. Cultural stigma and restrictive practices contributed to psychological distress and unsafe hygiene behaviours. These barriers were associated with adverse outcomes such as reproductive tract infections and increased school absenteeism. The quality appraisal indicated overall moderate to high methodological rigour, with most studies demonstrating acceptable quality despite some limitations related to study design and reporting.

**Conclusion:**

Menstrual health among tribal adolescent girls remains a significant public health concern in India. Addressing these challenges requires integrated, culturally sensitive interventions focusing on education, improved infrastructure, and equitable access to menstrual health services to enhance health outcomes and well-being.

## Introduction

Menstrual health is an essential aspect of adolescent well-being, but remains a neglected public health issue, especially in the case of marginalised populations like tribal adolescent girls in India. Menstruation is not just a biological process but also a socio-cultural phenomenon influenced by taboos, stigma, and lack of awareness. Challenges related to menstrual health management in tribal adolescent girls emanate from various barriers, including limited access to sanitary products, inadequate menstrual hygiene facilities, social taboos, and inadequate comprehensive education regarding reproductive health [1]. These barriers have to be dealt with in order to improve the health, education, and dignity of tribal adolescent girls in India. Tribal communities constitute 8.6% of India’s total population and mostly live in geographically isolated, socioeconomically poor conditions. The largest concentrations of Indigenous Peoples are found in the seven states of north-east India, and the so-called “central tribal belt” stretching from Rajasthan to West Bengal [2]. In these communities, Menstrual practices are influenced by local cultural beliefs and traditions, including restrictions on religious or social activities, limited discussion of menstruation, and the use of locally available materials when sanitary products are difficult to access or afford. Poverty, distance from markets and health facilities, and limited awareness can further contribute to inadequate menstrual hygiene practices and delayed treatment-seeking. Poor menstrual hygiene has been known to contribute to RTIs and other health problems, hence influencing the general health and school attendance of girls [3]. For example, among the Juang, a Particularly Vulnerable Tribal Group in Odisha, the cost and distance from markets were reported as barriers to sanitary-pad use, while cultural restrictions during menstruation were common and treatment-seeking for menstrual problems was low. These overlapping cultural, economic, and healthcare barriers highlight the need for interventions that are locally appropriate and sensitive to the specific circumstances of tribal communities [4].

Menstrual health management among tribal adolescent girls faces several challenges and barriers that impact their overall well-being, education, and social participation. A major issue is the lack of awareness and education about menstruation, leading to fear, anxiety, and misinformation. Limited reproductive health education in schools and cultural taboos within families further restrict open discussions, resulting in unsafe menstrual practices. Social stigma and cultural taboos also play a significant role in shaping negative perceptions of menstruation, often imposing restrictions on movement, religious participation, and diet. This stigma discourages girls from seeking support and essential resources. Additionally, access to sanitary products remains a significant challenge due to economic constraints and unavailability in remote areas, forcing many girls to rely on unhygienic alternatives, increasing their risk of infections. The absence of adequate water, sanitation, and hygiene (WASH) facilities further complicates menstrual hygiene management, contributing to school absenteeism and, in some cases, dropouts. Education is directly affected, as many girls miss school due to fear of leakage, lack of privacy, and inadequate menstrual hygiene facilities, widening gender disparities in literacy and future employment opportunities [5].

The Government of India has implemented several programmes to improve menstrual health and hygiene among adolescent girls. These include the Menstrual Hygiene Scheme (MHS) under the National Health Mission, which promotes menstrual hygiene awareness, access to sanitary products, and safe disposal among adolescent girls. The National Menstrual Hygiene Policy for School-Going Girls, provides a broader framework for access to menstrual hygiene products, menstrual health education, gender-responsive sanitation facilities, and environmentally safe disposal of menstrual waste [6]. In relation to tribal communities, broader tribal-focused programmes also have relevance to menstrual health. The Pradhan Mantri Janjati Adivasi Nyaya Maha Abhiyan (PM-JANMAN) was introduced for Particularly Vulnerable Tribal Groups (PVTGs) and includes interventions related to health and nutrition, safe drinking water, sanitation, education, housing, connectivity, and livelihoods [7]. Similarly, the Dharti Aaba Janjatiya Gram Utkarsh Abhiyan (DA-JGUA) aims to address infrastructure and human-development gaps in tribal-majority villages, including improving access to health, education, Anganwadi services, and livelihood opportunities [8]. Although PM-JANMAN and DA-JGUA are not specifically menstrual-health programmes, their focus on health, water, sanitation, education, and access to essential services is directly relevant to the barriers affecting menstrual health and hygiene among tribal adolescent girls. However, geographical remoteness, poverty, cultural beliefs, and limited healthcare infrastructure may affect the reach and effective implementation of these programmes among tribal adolescent girls. Therefore, understanding the specific barriers faced by these communities is important for developing more equitable and context-specific menstrual health interventions.

Furthermore, insufficient healthcare support and ineffective policy implementation in tribal areas limit access to gynaecological care and menstrual health education. While government schemes aim to address these issues, their reach and execution remain inadequate. Strengthening healthcare outreach, improving infrastructure, and integrating menstrual health education into primary healthcare services are crucial to overcoming these barriers. The objective of this review is to explore the key menstrual health challenges, barriers, and associated outcomes among tribal adolescent girls in India.

## Materials and methods

This systematic review is registered in PROSPERO with registration no: PROSPERO 2026 CRD420261369097.

This systematic review was conducted following the Preferred Reporting Items for Systematic Reviews and Meta-Analyses (PRISMA 2020) guidelines. The detailed PRISMA checklist were followed and it is mentioned in the supporting file (S1 PRISMA Checklist).

Research Question based on the PICO Framework), Population (P): Tribal adolescent girls in India, Exposure (I): Menstrual health challenges and barriers, Comparison (C): Not applicable, and Outcome (O): Menstrual hygiene practices, health outcomes, and associated determinants.

### Research question

What are the key menstrual health challenges and associated outcomes among tribal adolescent girls in India?

### Search strategy

A search strategy was developed in consultation with subject experts and refined iteratively to ensure sensitivity and specificity. The search strategy used the PICO framework, focusing on tribal adolescent girls in India and their menstrual health challenges. A comprehensive and systematic search was conducted across multiple electronic databases, including PubMed, Scopus, and Google Scholar. Key search terms related to menstrual health (such as “menstrual hygiene,” “menstrual health,” and “menstrual hygiene management”), population (such as “adolescent girls,” “adolescent,” and “teenagers”), and tribal Indian context (including “tribal,” “scheduled tribe,” “indigenous”, India, “North India”, “South India”, “East India”, and “West India”) were identified and combined using Boolean operators (AND, OR) to enhance sensitivity and specificity. Database-specific subject headings, including MeSH terms in PubMed, were incorporated where applicable. Grey literature was also explored by screening the first three pages of Google Scholar. Additionally, reference lists of included studies and relevant reviews were manually screened to identify any further eligible articles. The search was limited to studies published in English between January 2015 and March 2026. A preliminary search was done in PubMed, followed by Scopus. Details of the search strategy are mentioned in Table 1. The search strategy using Boolean operators OR & AND, databases included PubMed, Scopus and grey literature (Google Scholar).

**Table 1:** Search strategy using Boolean operators OR & AND, databases included PubMed, Scopus and grey literature (Google scholar).

Database	Population (P)	Exposure (I)	Comparison (C)	Outcome (O)
PubMed	“Adolescent”[MeSH], “Adolescent Health”[MeSH], “Students”[MeSH], girls, “Adolescent girls,” “Teenage girls,” youths, “Indigenous Peoples”[MeSH], Trib*, tribal, native, “Scheduled Tribes,” India	“Menstruation”[MeSH], “Menstrual Hygiene”[MeSH], “Hygiene”[MeSH], “Sanitary Napkins”[MeSH], “Menstrual Hygiene Products,” “Reproductive Health”[MeSH], “Health Knowledge, Attitudes, Practice”[MeSH], menstrual health, periods	Not Applicable	“Health Status”[MeSH], “Reproductive Tract Infections”[MeSH], “Menstrual Cycle Disorders”[MeSH], “Dysmenorrhea”[MeSH], “Premenstrual Syndrome”[MeSH], school absenteeism, awareness, accessibility, hygiene practices
Scopus	“Adolescent girls” OR “Teenage girls” OR girls OR youths AND Trib* OR Indigenous OR Native AND India OR “South India” OR “North India” OR “West India” OR “East India” OR “North East India” OR “East Central India” OR “Union territories”	“Menstrual Health” OR Menstruation OR “Menstrual Hygiene” OR Periods OR “Sanitary pads” OR “Sanitary napkins” AND Challenges OR Barriers OR Hurdles OR Obstacles OR Issues OR Complications OR Difficulties	Not Applicable	Menstrual hygiene practices, health outcomes, awareness, accessibility, school absenteeism
Google Scholar	“Adolescent girls” OR “Teenage girls” OR girls OR youths AND Trib* OR Indigenous OR Native AND India OR “South India” OR “North India” OR “West India” OR “East India” OR “North East India” OR “East Central India” OR “Union territories”	“Menstrual Health” OR Menstruation OR “Menstrual Hygiene” OR Periods OR “Sanitary pads” OR “Sanitary napkins” AND Challenges OR Barriers OR Hurdles OR Obstacles OR Issues OR Complications OR Difficulties	Not Applicable	Menstrual hygiene practices, health outcomes, awareness, accessibility, school absenteeism

### Eligibility criteria

Studies were selected based on predefined eligibility criteria. Both quantitative study designs, including cross-sectional, interventional, and randomised controlled trials, as well as qualitative approaches such as focus group discussions, in-depth interviews, and perspective-based studies, were included to ensure a comprehensive understanding of the research topic. Grey literature was also considered by screening the first three pages of Google Scholar, and relevant references cited within systematic reviews were examined for additional eligible studies.

Studies that did not report primary data, such as editorials, narrative reviews, and abstracts without full-text availability, were excluded. Only articles published in the English language were considered. To enhance the completeness of the search, the reference lists of all included studies and relevant reviews were manually screened to identify any additional studies that may have been missed during database searching.

The review was restricted to studies published between January 2015 and March 2025, as this period follows the implementation of the Rashtriya Kishori Swasthya Karyakram (RKSK) in 2014, which marked a significant policy shift in prioritising menstrual health within adolescent healthcare in India.

### Study selection

With the help of keywords, citations were retrieved and imported to the Rayyan software [9]. A systematic review web-based management system [10]. Titles and abstracts screening was done by the three reviewers, RR, JB, and SIA, independently. In case of any discrepancy, opinion was taken from the fourth reviewer, SIA. Full text screening followed by the development of a data extraction sheet was done independently by three reviewers, RR, JB, and MP, of the studies retrieved from the title/abstract screening. The included studies underwent charting of the data for the final review. Reporting details are mentioned in a PRISMA 2020 flowchart (Fig 1) [11]. Studies were included based on the inclusion criteria. Table 2: Data reporting chart depicting author/year, title of the study, population, study design, and outcome.

**Table 2:** Data reporting chart depicting author/year, title of the study, population, study design, and outcome.

Sl.No.	Author, year	Title of the Study	Population	Study region	Exposure (I)	Outcome	Quality Assessment as per JBI format (paper included: Yes/No)
1	Chauhan, S. et al, 2016	Examining the predictors of use of sanitary napkins among adolescent girls: A multi-level approach	Adolescent girls (10–19 years)	Uttar Pradesh, Bihar	Socio-demographic factors, education, and access to sanitary napkins	Use of Sanitary Napkins	Moderate (Yes)
2	Sharma, S. et al, 2021	Comparing Reproductive Health Awareness, Nutrition, and Hygiene among Early and Late Adolescents from Marginalised Populations of India: A Community-Based Cross-Sectional Survey	Adolescent girls (10–19 years)	East, West, North, South, and Central India	Socioeconomic status, nutrition, and hygiene awareness	Unsafe practising	Moderate (Yes)
3	Gold-Watts, A et al, 2020	A qualitative study of adolescent girls’ experiences of menarche and menstruation in rural Tamil Nadu, India	Adolescent girls (10–19 years)	Tamil Nadu	Cultural beliefs, experiences of menarche	Positive experience, Distressing experience, restriction	High (Yes)
4	Anand, E et al, 2015	Menstrual hygiene practices and its association with reproductive tract infections and abnormal vaginal discharge among women in India	Female age group 15–49 years	28 states and 6 union territories	Menstrual hygiene practices	Safe practise	High (Yes)
5	Chattopadhyay, A et al, 2019	WASH practices and its association with nutritional status of adolescent girls in poverty pockets of eastern India	Adolescent girls (10–19 years)	Bihar, Odisha, and Chhattisgarh	WASH practices, poverty	Unsafe practising (no use of sanitary napkins)	Moderate (Yes)
6	Singh, A et al, 2022	Menstrual hygiene practices among adolescent women in rural India: a cross-sectional study	Women of reproductive age group (15–49 years)	28 states and 7 union territories	Education, socioeconomic status	Less education less menstrual hygiene practises	High (Yes)
7.	D. A. Tan et al, 2017	Cultural aspects and mythologies surrounding menstruation and abnormal uterine bleeding	Women of reproductive age group (14–45 years)	Round the world, including India	Cultural beliefs, myths	Lack of awareness of menstrual hygiene	Moderate (Yes)
8.	Kathuria, B et al, 2018	Effects of Socio-Economic Conditions on Usage of Hygienic Method of Menstrual Protection among Young Women in EAG States of India	Women (15–24 Years)	Empowered Action Group States (EAG), India	Socioeconomic conditions, caste	Lack of Menstrual Protection among Schedule caste	Moderate (Yes)
9.	Kumari, S et al, 20211	Knowledge and practices related to menstruation among tribal adolescent girls	3-18 years adolescent girls	Munda tribe, Khunti, Jharkhand	Knowledge sources (mother, peers)	Aware of Menstrual hygiene through mother & friends	Moderate (Yes)
10.	Bhagwat, A et al,	A Psychosocial Lens on an Indigenous Initiative to Address Menstrual Health and Hygiene in Indian Villages	School adolescent girls	Gujarat	Psychosocial and community interventions	Poor menstrual hygiene practices	Moderate (Yes)
11.	Ram, U et al, 2020	Factors Associated with Disposable Menstrual Absorbent Use Among Young Women in India	15-24 Years age group women	India	Socioeconomic status, caste	SC/ST have low use of absorbent napkins	High (Yes)
12.	Sakshi, S et al, 2024	Examining the impact of socioeconomic and demographic factors on women’s health: A survey-based study of menstrual hygiene, pregnancy, and post-pregnancy care in the Gonds Tribe of Sonbhadra, Uttar Pradesh	Female 15–60 years	Gonda tribe, Sonbhadra, Uttar Pradesh	Socioeconomic & demographic factor	Use of Cotton clothes	Moderate (Yes)
13.	P, Aparnavi et al, 2024	Suitability, acceptability, feasibility of modern menstrual methods: a qualitative study in Coimbatore district, Tamil Nadu, India	15-49 Years women	Tamil Nadu, India	Acceptability of menstrual products	Use of cotton clothes by tribal women	High (Yes)
14.	Panda, N et al, 2024	Menstrual health and hygiene amongst adolescent girls and women of reproductive age: a study of practices and predictors, Odisha, India	10-49 years female	Odisha, India	Caste, awareness, socioeconomic factors	Menstrual hygiene awareness is more in the General caste	High (Yes)
15.	Thakur, J et al, 2020	Understanding menstrual characteristics from the perspective of reproductive energetics: a study on the adolescent Oraon tribal populations	10-19 years adolescent girls	Oraon tribes, Jalpaiguri, West Bengal	Biological and cultural factors	Irregularity in periods	Moderate (Yes)
16.	Vayeda, M et al, 2021	Improving menstrual hygiene management among adolescent girls in tribal areas of Gujarat: an evaluation of an implementation model integrating the government service delivery system	Adolescent girls 10–19 years	Narmada district, Gujarat, India	Intervention (government service delivery model)	Safe Menstrual hygiene methods	High (Yes)
17.	Bhoi, N et al, 2024	Health status of particularly vulnerable tribal groups (PVTGs) of Odisha: a narrative review	Any age group and gender	Odisha, India	General tribal health determinants	Use of old clothes	Low (No)
18.	Chatterjee, R et al, 2024	Menstrual disorders and associated factors among rural and tribal adolescent girls in India: A systematic review and meta-analysis	10-19 years adolescent girls	India	Multiple determinants (taboos, lifestyle)	Social taboos, lack of physical activities, and menstrual irregularities.	Low (No)
19.	Mudi, K, P et al, 2023	Menstrual health and hygiene among Juang women: a particularly vulnerable tribal group in Odisha, India	Married women 15–49 age group,	Keonjhar district of Odisha, India	Cultural practices, socioeconomic status	Use of old clothes	Moderate (Yes)
20.	Karjee, S et al, 2023	Contextualizing the socio-economic and spatial patterns of using menstrual hygienic methods among young women (15–24 years) in India: A cross-sectional study using the nationally representative survey	Young women 15–24 years	Assam, Bihar, Chhattisgarh, Jharkhand, Madhya Pradesh, and Rajasthan	Wealth, autonomy, social media exposure	Safe Menstrual hygiene method, awareness through mass social media, autonomy, and household wealth.	High (Yes)
21.	Meher, T et al, 2023	Dynamics of usage of menstrual hygiene and unhygienic methods among young women in India: a spatial analysis	15-24 Year age group	India	Spatial and socioeconomic factors	Unsafe menstrual hygiene practice.	High (Yes)
22.	Vishwakarma, D et al, 2021	Interlinking menstrual hygiene with Women’s empowerment and reproductive tract infections: Evidence from India	15-24 Year age group	India	Empowerment, hygiene practices	Utilisation of hygienic method	High (Yes)
23.	Babbar, K et al, 2024	Bleeding at the Margins: Understanding Period Poverty among SC and ST Women Using Decomposition Analysis	15-24 years	India	Caste-based inequality, economic status	Inequalities in period products	High (Yes)
24.	Agarwal, V et al, 2017	Menstrual Hygiene: Knowledge and Practice among Adolescent Girls of Rural Sabarkantha District	13-18 years adolescent girls	Sabarkantha, Maharashtra	Knowledge and awareness	Safe hygiene practice	Moderate (Yes)
25.	Borkar, SK et al, 2022	Study of Menstrual Hygiene Practices Among Adolescent Girls in a Tribal Area of Central India	10-19 years	Nagpur, Maharashtra	Cultural beliefs, awareness	Awared, safe hygiene practices, and cultural beliefs	Moderate (Yes)
26.	Kakeri, M et al, 2018	Knowledge and Practice Gap for Menstrual Hygiene among Adolescent School Girls of the Tribal District of Maharashtra, India: A Cross-Sectional Study	Adolescent girls, 12–16 years	Palghar, Maharashtra	Knowledge gap, education	Need awareness	Moderate (Yes)
27.	Udayar, ES et al, 2022	Prevalence and Predictors of Dysmenorrhea and its Impact on Quality of Life among Tribal Adolescent Girls in India	Adolescent girls 10–19 years	Chittoor, Andhra Pradesh	Dysmenorrhea predictors (biological, lifestyle)	School absenteeism	High (Yes)
28.	Raj, R et al, 2026	Multi-stakeholder perspectives on reproductive and adolescent healthcare schemes in tribal regions of India: A qualitative study	Adolescent girls & tribal women in the reproductive age group 10–49 years	East Singhbhum, Jharkhand	Barriers and enablers in reproductive and adolescent healthcare delivery (e.g., awareness, infrastructure, governance, cultural practices)	Low awareness of schemes, absenteeism of healthcare workers, inadequate infrastructure, cultural reliance on traditional medicine, financial and transport barriers, delayed incentives, limited use of health technology; enablers include community support, supervision, and NGO collaboration	High (Yes)

**Fig 1:**
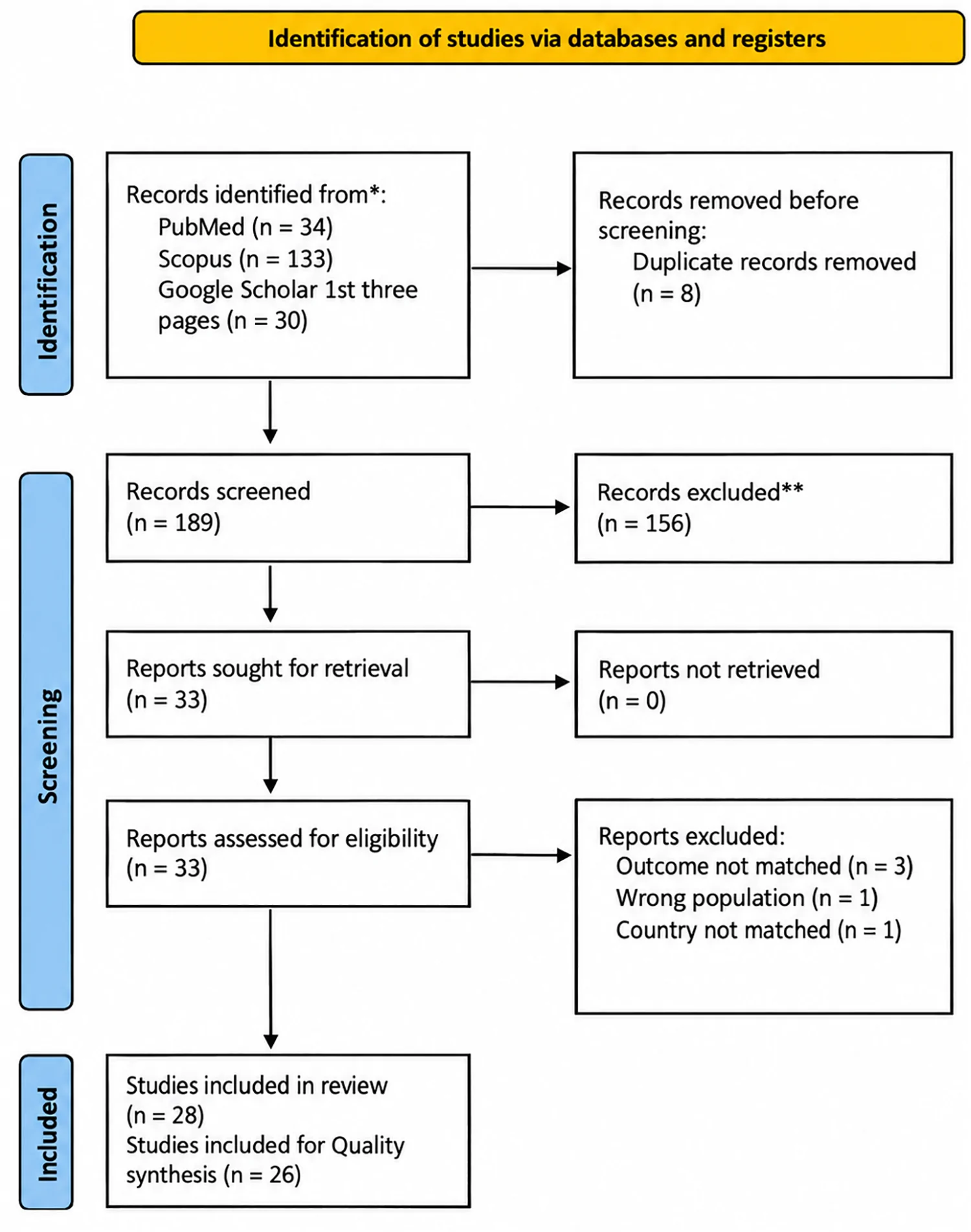
PRISMA 2020 flowchart.

### Data extraction

A standardised data extraction form was developed and utilised to collect relevant information from each included study systematically. This included key study characteristics such as the year of publication, geographical location, and study design, along with details of the study sample, including population characteristics and sample size. Additionally, important findings related to menstrual health, including identified challenges, practices, and associated factors, were extracted to facilitate comprehensive analysis and synthesis. Data extraction was done by RR, JB, and VLR.

### Quality synthesis

The methodological quality of the included studies was evaluated using the Joanna Briggs Institute (JBI) critical appraisal tools, with specific checklists applied according to the respective study designs. This assessment enabled a systematic evaluation of potential biases and overall study rigour. Based on the appraisal outcomes, studies were classified into three categories: low risk of bias, moderate risk of bias, and high risk of bias, to ensure transparency and reliability in the synthesis of evidence. Two researchers RR and VLR did the synthesis, conflicts were resolved by the third researcher MP. A detailed characteristics and quality synthesis of the studies are mentioned in the supporting file (S2 Data extraction sheet in S1 PRISMA Checklist).

### Data synthesis

Due to the substantial heterogeneity observed across the included studies in terms of study designs, populations, outcome measures, and reported variables, a meta-analysis was not considered appropriate. Instead, a narrative thematic synthesis approach was adopted to systematically integrate and interpret the findings. Initially, data extracted from individual studies were organised and summarised in tabular form to facilitate comparison. Subsequently, an inductive approach was used to identify recurring patterns, concepts, and relationships within the data.

The findings were then grouped into key thematic domains, such as socioeconomic determinants, cultural barriers, accessibility of menstrual products, health risks, regional disparities, and school-related outcomes. These themes were developed through an iterative process involving multiple reviewers to enhance consistency and reduce subjective bias. Where appropriate, results from quantitative and qualitative studies were triangulated to provide a more comprehensive understanding of menstrual health challenges among tribal adolescent girls. This approach allowed for the synthesis of diverse evidence while preserving the contextual richness of individual studies.

## Results

A total of 197 retrievals were identified through database searching alone: PubMed-34, Scopus-133, and Google Scholar-30. Following the screening process, a total of 28 eligible studies were included in the synthesis. The detailed study selection is depicted in the PRISMA-SR flowchart, Fig 1: PRISMA 2020 flowchart [11]. The following themes were generated from the evidence shown in Fig 2: Six themes generated from the evidences.

**Fig 2:**
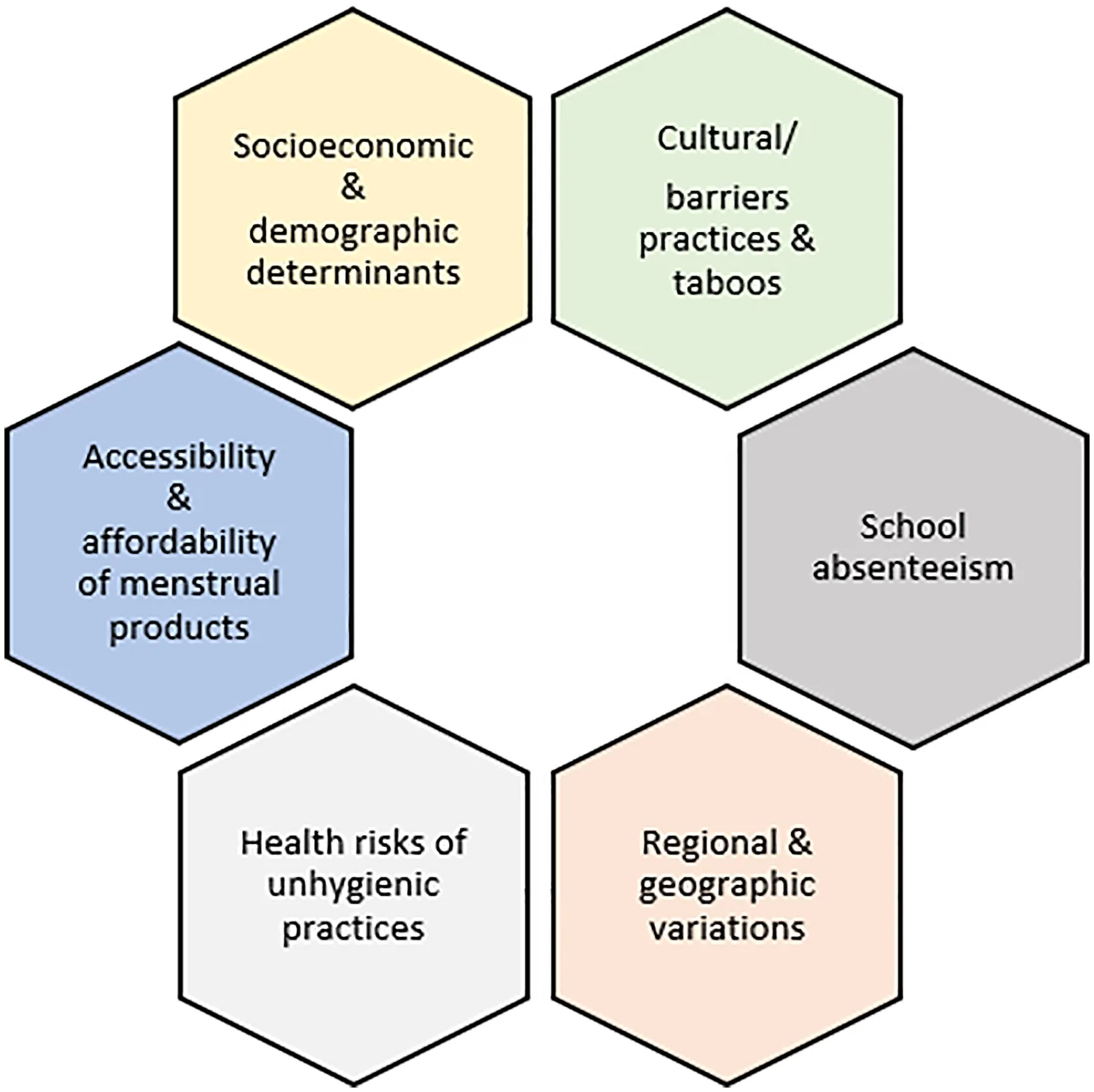
Six themes generated from the evidences.

1. Socioeconomic and demographic determinants:

The more educated the adolescent girl and her mother, the higher the use of sanitary napkins. Wealth and economic status, such as those from richer households, are significantly more likely to use sanitary pads than those from poorer households. Exposure to social media is a determinant of menstrual hygiene practice, making sanitary napkin use more likely. Caste and Social Stratification reported that Scheduled Tribe (ST) and Scheduled Caste (SC) women have lower odds of using hygienic menstrual methods compared to General category women. Sanitary napkin use is more prevalent in urban areas than in rural areas. Low awareness of health schemes, including menstrual health, language barriers, and poor outreach in tribal communities, further exacerbate these socioeconomic disparities, limiting access to menstrual health information and services.

2. Cultural/barriers, practices and taboos

There are some restrictions on family contact or religious activities, cooking, and not entering temples during menstruation. Menstruation-Based Fears and Anxiety, myths of attracting wild animals and ghosts possessing the woman while menstruating, cause psychological suffering, an initiatory rite and a social experience at menarche. In a few communities, menarche is observed through initiation rituals, and this rite brings support for social constructs like gender, even showing that the girl is now ready for marriage.

3. Accessibility and affordability of menstrual products

Many women, particularly in tribal and economically weaker sections, find sanitary pads unaffordable and continue to use cloth. Limited market access, such as distance from markets, restricts access to menstrual hygiene products, especially in remote tribal areas. Government and NGO interventions, including kishori group meetings and school-based awareness programs, have been effective in improving menstrual hygiene knowledge and practices.

4. Health risks associated with unhygienic practices

The use of unhygienic absorbents is associated with RTIs and other menstrual health problems. The impact of sanitation facilities, with the availability of household toilets, enhances the use of sanitary menstrual practices. Nutritional and lifestyle factors: intake of diet, BMI, and physical activity have been related to menstrual disorders like dysmenorrhea and premenstrual syndrome (PMS).

5. Regional & geographic variations

The included studies represented diverse geographical settings across India, including Uttar Pradesh, Bihar, Jharkhand, Odisha, Chhattisgarh, Gujarat, Maharashtra, Tamil Nadu, Andhra Pradesh, West Bengal, and Assam, as well as studies using nationally representative datasets. The evidence therefore covered both eastern, central and populations from northern, western, southern, and northeastern India. Considerable geographical variation was observed in menstrual hygiene practices, access to sanitary products, water and sanitation facilities, menstrual knowledge, and healthcare access. Sanitary napkin use was generally higher in southern states, whereas lower levels were reported in northern and central states, including Uttar Pradesh and Bihar.

6. School absenteeism and educational impact

The results indicate that menstrual pain is a major issue among students, with many choosing self-management strategies such as ignoring the pain or taking bed rest. This means that dysmenorrhea might be one of the key reasons for school absenteeism since discomfort may cause the student to avoid going to school. The very low percentage of adolescent girls who report seeking help from teachers (14.7%) may indicate a gap in awareness and support systems within schools regarding menstrual health [12]. This lack of guidance and assistance may further strengthen absenteeism, thus calling for better menstrual health education and support mechanisms in school settings. Inadequate training of frontline workers and a lack of adolescent-friendly services may contribute to insufficient support for menstrual health within educational and community settings.

### Quality synthesis

The quality appraisal of the included studies indicates an overall moderate to high methodological rigour across the evidence base. The majority of studies (n = 17) were rated as high quality, demonstrating robust study designs, adequate sample representation, and clear reporting of outcomes, particularly in large-scale cross-sectional and nationally representative analyses. A substantial proportion of studies (n = 10) were assessed as moderate quality, mainly due to limitations such as reliance on self-reported data, restricted geographic coverage, or limited control for confounding variables. Only two studies were rated as low quality and were subsequently excluded from the final synthesis, reflecting concerns related to methodological weaknesses and limited reliability of findings. Overall, the included evidence is reasonably strong, with most studies meeting acceptable quality standards; however, variations in study design, population heterogeneity, and contextual differences across regions should be considered while interpreting the results.

## Discussion

Menstrual health management (MHM) remains an important public health and social concern, particularly among adolescent girls and women from marginalised communities in India. The evidence base included in this review, largely comprising moderate to high-quality studies, consistently demonstrates significant disparities in menstrual hygiene. Many studies established the influence of age and educational factors regarding menstrual hygiene practices. A study by Chauhan et al. (2016) conducted among adolescents in Bihar and Uttar Pradesh reported that older adolescents (15–19 years) were more likely to use sanitary napkins than younger adolescents (10–14 years), partly attributed to higher educational attainment [13]. Similarly, Singh et al. (2022) reported that rural girls across India had significantly higher odds of exclusively using hygienic practices [10]. Another study done by Vayeda et al. (2022) concluded that education creates awareness and equips people with the knowledge of making appropriate choices in Gujarat [14]. Moreover, one of the studies stated that only a few girls knew about menstruation before menarche, thus emphasising the need for complete menstrual health education. However, the quality synthesis: moderate-quality studies indicate persistent gaps in awareness. Supporting these studies stated that menstrual awareness before menarche remains low [15,16].

In terms of socioeconomic and caste inequities in menstrual hygiene practices, there was a constant report of caste and socioeconomic status-based inequities in menstrual hygiene practices [17]. Hygienic menstrual methods were found to be used by approximately 77% of women, including adolescent girls in India, though variations exist across the socio-cultural and geographic dimensions. Factors such as education level, social status, and household wealth have a meaningful influence on hygienic menstrual practices. Studies found that girls from non-marginalised groups had a significantly higher odds of practising safe menstrual hygiene than those from Scheduled Castes (SC) and Scheduled Tribes (ST) [18,19]. A similar study by Anand et al. (2025) noted that women from tribal communities in India were more likely to use hygienic methods than SC women [20]. In one of the studies Singh et al. (2022), further reported that few ST women exclusively used hygienic methods compared to women from the general category [10]. This disparity highlights the need for targeted interventions addressing the menstrual health needs of marginalised communities. Studies found that adolescents from SC/ST backgrounds, poorer households, and those without access to water within household premises were significantly less likely to use sanitary napkins [21]. These findings are supported by Kathuria (2021), they reported that adolescents from the wealthiest category were almost eight times more likely to use sanitary methods than those from the poorest category [22]. Similar study found that economic factors, such as bank account ownership and mobile phone usage, influenced the adoption of hygienic menstrual practices. In other studies such as Vishwakarma et al. (2021), Chauhan et al. (2021), and Singh et al. (2022), adolescents with exposure to social media showed a higher preference for sanitary napkins in other studies [10,13,23]. As the study establishes, the social media is a major predictor of menstrual hygiene. Increased access to social media allows for normalisation in the conversation, and it can break the many myths and taboos against menstruation [22]. Educational interventions and improved social media exposure have been linked to better menstrual hygiene practices, though barriers such as affordability, lack of washing facilities, and concerns over waste disposal persist [24]. Addressing these barriers through targeted awareness programs and improved accessibility to hygienic menstrual products is crucial in improving menstrual health outcomes [25].

Community involvement also has a very significant role. Our review showed evidence from high-quality intervention studies demonstrates that leveraging government systems and community platforms improves knowledge and practices. Similarly, another study showed that girls who attended Kishori (adolescent) group meetings had higher odds of using sanitary napkins [21]. Studies also showed that it is possible to improve menstrual health knowledge and practices in tribal communities by using existing government systems. Such findings have underlined the necessity of wide-ranging community education programs to facilitate education on menstrual hygiene [14]. Sanitary pads are the most commonly used method, and cloth remains a popular alternative, especially among tribal women [26].

The findings demonstrate substantial geographical variation in menstrual health challenges among tribal adolescent girls in India. Studies from Uttar Pradesh and Bihar reported comparatively lower use of sanitary napkins and greater barriers related to affordability, access, and menstrual hygiene practices [13,21]. Whereas studies from southern India, particularly Tamil Nadu, indicated relatively higher use of hygienic menstrual products [26,27]. Evidence from eastern and central India, including Odisha, Jharkhand, Chhattisgarh, and West Bengal, further highlighted the influence of cultural practices, limited infrastructure, and restricted access to menstrual health information and services [17,21,28–30]. Studies from Gujarat and Maharashtra also identified gaps in awareness, affordability, and access to appropriate menstrual health resources [14,16,31]. The geographical differences should be interpreted in relation to the social, cultural, economic, and health-system contexts of tribal communities. For example, studies among tribal populations in Odisha documented culturally specific menstrual restrictions and beliefs, while evidence from Jharkhand highlighted challenges related to access to information and healthcare services [28,30]. Similarly, findings from Gujarat and Maharashtra suggest that availability of services and awareness may influence menstrual hygiene practices. These variations indicate that, although menstrual health barriers are common across tribal populations, their form and intensity differ by state and local context. Therefore, interventions should be geographically and culturally responsive rather than relying on a uniform approach across tribal communities in India.

Cultural taboos, restrictions, and psychological impact regarding menstruation have subsided with time, and no relevant activities are left untouched. The reviewed studies reveal a wide set of restrictive practices that influence the psychological well-being of adolescent girls. The painful experiences during menstruation, such as exclusion from religious spaces, restriction while cooking, and segregation within the household, were widely reported in some studies [19]. They observed that Juang and Dongria Kondh tribal women from Odisha experienced a lot of restrictions, including social exclusion and avoiding religious activities, during menstruation. Superstitions further increase menstrual distress [4,30]. However, girls were said that improper disposal of menstrual absorbents for menstruation would attract wild animals, while others think ghosts haunt women during menstruation [19]. All these myths make one feel fear, anxiety, and isolated, and thus impact one’s mental health.

The study brings out both the positive and distressing experiences related to menstruation. Cultural celebrations, such as menarcheal rites, play a very significant role in reinforcing positive menstrual experiences since they symbolise a girl’s transition to womanhood. These celebrations also reinforce societal expectations, such as early marriage, which is very restrictive. Another contributing factor to distress is menstrual taboos and myths. Many girls associate menstruation with fear, stress, and psychological distress due to cultural beliefs [16]. Myths about menstrual absorbent disposal attracting wild animals or menstruating women being haunted by ghosts exacerbate these concerns, leading to restrictive behaviours such as sleeping outdoors or carrying protective objects like copper nails [27]. Furthermore, restrictive practices, including being segregated, refraining from cooking, and being barred from religious spaces, are still prevalent [32]. Menstrual hygiene practices also vary significantly, with many women, particularly from marginalised communities, having limited access to disposable absorbents. Exclusive use of disposable products is influenced by caste, socioeconomic status, and education level, with women from Scheduled Castes, Scheduled Tribes, and Other Backwards Classes having lower odds of using such products [27,33]. Hygiene awareness remains inadequate among tribal women, where primitive practices, such as tying a leaf with mud around the waist or sitting on an earthen pot, persist, leading to infections and discomfort [32]. Findings from a qualitative study done by Raj et al. (2026), also highlight key barriers to menstrual health in tribal areas of Jharkhand, including low awareness, cultural beliefs, poor infrastructure, and gaps in healthcare delivery. These findings support the present review, emphasising the need for stronger health systems, targeted education, and community-based interventions [34].

Poor infrastructure remains among the biggest challenges to menstrual hygiene management. Chattopadhyay et al. (2019),reported that lack of access to water within household premises and lack of sanitation facilities were the two major contributors to unhygienic menstrual practices in the states such as Bihar, Odisha, and Chhattisgarh [21]. Similarly, Singh et al. (2022), found major geographic disparities, with Tamil Nadu reporting maximum exclusive hygienic method use compared to Uttar Pradesh [10]. Meher et al highlighted that Madhya Pradesh and Bihar were unhygienic menstrual practices hotspots due to poverty, lack of toilet facilities, and rural residence [35]. The issue also lies in the availability of menstrual products. One study indicated that only a small proportion of rural women in Haryana used sanitary napkins, and financial constraints were the prime barrier. The mean price women were willing to pay per napkin was Rs. 0.54 (US$ 0.01), which meant that there is a need for cost-effective and subsidised menstrual products [28,36].

The consequences of inadequate menstrual hygiene are not limited to social and psychological distress but also have severe health risks. Vishwakarma et al. (2021), found that lack of proper menstrual hygiene is related to increased risks of reproductive tract infections (RTIs) [23]. Another study also stressed the fact that dysmenorrhea, irregular menstruation, and premenstrual syndrome (PMS) were common among women with poor menstrual hygiene [37]. Additionally, Thakur et al. (2020), found an association between dietary habits, body fat percentage, and menstrual irregularities, thus suggesting the need for integrated health interventions that include nutrition education [29].

The policy and programmatic interventions addressing menstrual hygiene issues should be multi-layered, involving education, infrastructure development, community engagement, and policy interventions. A similar study demonstrated the impact on MHM in tribal girls through the effective use of government systems in enhancing knowledge and practice [14]. Such initiatives, if extended to rural or marginalised communities, would help in filling gaps in menstrual health. Menstrual health awareness should be actively promoted by functionaries of the health sector. Delfin et al. opined that healthcare workers should be involved in menstrual education programmes [36]. In the meanwhile, one study opined that the girl students should be given guidance in schools and menstrual-related fears and anxieties can be removed through health camps [37]. Affordability and accessibility of menstrual products should be prioritised. Government initiatives like the distribution of subsidised sanitary napkins can really enhance menstrual hygiene practices among low-income and marginalised communities. Additionally, incorporating menstrual hygiene education into school curricula can help dismantle deep-rooted taboos and myths surrounding menstruation. Studies also reported that nearly all participants ignored menstrual pain, while a few opted for bed rest, and resorted to self-medication, some used hot applications and relied on home remedies. Around 40% managed dysmenorrhea independently, one-fourth sought support from friends, and reached out to teachers for help [12]. Hence, restrictions such as exclusion from religious functions and school remain prevalent [28]. These findings highlight a predominant tendency for self-management, with limited external assistance.

Our review highlights that menstrual health challenges among tribal adolescent girls are driven by intersecting socioeconomic, cultural, and infrastructural barriers, resulting in poor hygiene practices, health risks, and educational disruptions. The findings emphasise that addressing these outcomes requires integrated, context-specific interventions focusing on education, accessibility, and health system strengthening through inclusion of menstrual health competencies in school education, better access to affordable menstrual products, and strengthened WASH infrastructure. Targeted interventions like street plays, quizzes, poster competitions, rallies, fundraising through sports and literary events, which can be accessible to tribal girls, should address socioeconomic and caste disparities, while community engagement and culturally sensitive approaches are essential to reduce stigma. Strengthening frontline healthcare services and promoting adolescent-friendly programs are crucial. Future research should focus on high-quality, longitudinal, and intervention-based studies covering diverse tribal populations.

### Study limitations

This systematic review has several limitations that should be considered while interpreting the findings. First, the review included studies published only in English, which may have led to language bias and exclusion of relevant regional literature. Second, the majority of included studies were cross-sectional in design, limiting the ability to establish causal relationships between menstrual health determinants and outcomes. Third, there was considerable heterogeneity across studies in terms of study populations, geographic regions, methodologies, and outcome measures, which restricted the possibility of conducting a meta-analysis and limited comparability. Additionally, qualitative studies relied on self-reported data, which may be subject to recall and social desirability bias. Some studies had limited geographic coverage, with underrepresentation of certain tribal groups and regions, affecting generalizability. Furthermore, variations in quality, with a proportion of studies rated as moderate, indicate potential methodological limitations such as inadequate control of confounders. Lastly, the review focused primarily on published literature, and despite limited grey literature inclusion, there remains a possibility of publication bias.

## Conclusion

This systematic review identified key menstrual health challenges among tribal adolescent girls in India, leading to various outcomes shaped by contextual factors such as socioeconomic status, cultural beliefs, practices, and infrastructural factors. The findings highlight low awareness before menarche, inequitable access to hygienic products, and persistent caste and economic disparities that hinder safe practices. Cultural taboos and stigma contribute to psychological distress and poor health-seeking behaviours, while inadequate WASH facilities and limited healthcare support further restrict effective menstrual hygiene management. These challenges lead to increased health risks, including reproductive tract infections, and higher school absenteeism, affecting both health and education. Overall, menstrual health remains a neglected public health issue, requiring integrated, culturally sensitive interventions to improve outcomes and ensure dignity and well-being.

## Supporting information

S1 PRISMA Checklist This S1 file contains PRISMA 2020 checklist used for reporting the systematic review and meta-analysis. S2 data extraction sheet.xlsx. This S2 file contains a data extraction sheet containing the information extracted from the studies included in the systematic review and meta-analysis.
(DOCX)
