# Supplementary material for: Menstrual health challenges and associated barriers among tribal adolescent girls in India: A systematic review
Source: PLoS One. 2026 Oct 12;21(10):e0360401. doi: 10.1371/journal.pone.0360401 (PMC13657302; doi:10.1371/journal.pone.0360401)
Supplement: Supplementary file 2 [file pone.0360401.s003.docx]

**Reviewer #1:** A very interesting review of a relatively neglected area of discussion at a wider level. I think the paper is well written, well structured, and the review has followed a rigorous process. I have some reflections which would help to strengthen the paper further.

1. In the introduction, it may be worth adding some detail about the policy context for MHH in India, e.g. SBM and the various national schemes in place to set the tone, and to help to position the paper's focus on tribal communities. There is reference to tribal communities as making up 8.6% of India's population. Given that tribal communities are such a small percentage and in isolated areas, can you please expand a little more on the details about the cultural practices, poverty and lack of healthcare services and the impact this has on them?

**Answer:** Thank you for this valuable suggestion. We have updated the we have expanded the Introduction to provide greater context on India’s policy landscape for menstrual health and hygiene (including SBM and relevant national schemes), and to elaborate on the cultural practices, poverty, limited healthcare access, and related challenges affecting tribal communities.
2. The paper refers to government schemes for tribal populations at the top of page 10. It may be worth clarifying which government schemes have been put in place already, or at least to give some examples in relation to tribal communities.

**Answer:** Thank you for this important remark. We have rephrased the paragraph and added similar government schemes.

3. I appreciate the strength of the well-designed search strategy. Did the search also specify "India" as a term?

**Answer:** Yes, we have mentioned “India” as a term in the search strategy and updated in the search terms used in the manuscript.

4. An overall bit of feedback for the results section: as this is a review, I would reference each publication throughout the whole section. What was classed as 'media' as part of the review- was social media included? It may be worth specifying this more clearly. In the section on school absenteeism, a reference is needed for the 14.7% statistic on teachers.

**Answer:** Thank you for this helpful feedback; we have revised the Results section to consistently cite the included publications, clarified that “media” included social media where applicable, and added the appropriate reference for the reported 14.7% statistic on teachers.

5. I would urge caution about saying MHM is the critical issue for public health in India, and maybe loosen the language at the start of the discussion section.

**Answer:** Thank you for this important observation. We agree that describing menstrual health management (MHM) as “the critical issue” in India may overstate the evidence presented in our review. We have therefore revised the opening of the Discussion to use more cautious and evidence-based language. Specifically, the phrase “The critical issue in India's public health” has been replaced with “Menstrual health management (MHM) remains an important public health and social concern, particularly among adolescent girls and women from marginalised communities in India.” This revision better reflects the scope and findings of our review while avoiding an overly broad national claim.

6. Throughout the text, it might be worth adding the geographies/states the studies are focused on, to deepen the understanding of the issues in different contexts in India. This would make it more relatable and easier to understand the findings.

**Answer:** We have updated as per the suggestion in the results and discussion section. State and geographical location.

7. I would also recommend you clarify the language a little throughout the discussion - repeated references to 'studies' is confusing, and I would advise changing to "[name] et al found that...."

Answer: We have updated the changes as per suggested.

Overall, this is a thorough and important review on a niche and neglected area in MHH in India.
