# Supplementary material for: Menstrual health challenges and associated barriers among tribal adolescent girls in India: A systematic review
Source: PLoS One. 2026 Oct 12;21(10):e0360401. doi: 10.1371/journal.pone.0360401 (PMC13657302; doi:10.1371/journal.pone.0360401)
Supplement: Peer review history

# Peer review: Decision Letter 0

**DOI:** 10.1371/journal.pone.0360401.r001

**Authors:** Alison Parker

7 Sep 2026

Dear Dr. Begum,

Thank you for submitting your manuscript to PLOS One. After careful consideration, we feel that it has merit but does not fully meet PLOS One’s publication criteria as it currently stands. Therefore, we invite you to submit a revised version of the manuscript that addresses the points raised during the review process.

Please submit your revised manuscript by Oct 07 2026 11:59PM. If you will need more time than this to complete your revisions, please reply to this message or contact the journal office at plosone@plos.org. When you're ready to submit your revision, log on to https://www.editorialmanager.com/pone/ and select the 'Submissions Needing Revision' folder to locate your manuscript file.

- A letter that responds to each point raised by the academic editor and reviewer(s). You should upload this letter as a separate file labeled 'Response to Reviewers'.
- A marked-up copy of your manuscript that highlights changes made to the original version. You should upload this as a separate file labeled 'Revised Manuscript with Track Changes'.
- An unmarked version of your revised paper without tracked changes. You should upload this as a separate file labeled 'Manuscript'.

If you would like to make changes to your financial disclosure, please include your updated statement in your cover letter. Guidelines for resubmitting your figure files are available below the reviewer comments at the end of this letter.

If applicable, we recommend that you deposit your laboratory protocols in protocols.io to enhance the reproducibility of your results. Protocols.io assigns your protocol its own identifier (DOI) so that it can be cited independently in the future. For instructions see: https://journals.plos.org/plosone/s/submission-guidelines#loc-laboratory-protocols. Additionally, PLOS One offers an option for publishing peer-reviewed Lab Protocol articles, which describe protocols hosted on protocols.io. Read more information on sharing protocols at https://plos.org/protocols?utm_medium=editorial-email&utm_source=authorletters&utm_campaign=protocols.

As the corresponding author, your ORCID iD is verified in the submission system and will appear in the published article. PLOS supports the use of ORCID, and we encourage all coauthors to register for an ORCID iD and use it as well. Please encourage your coauthors to verify their ORCID iD within the submission system before final acceptance, as unverified ORCID iDs will not appear in the published article. *Only* the individual author can complete the verification step; PLOS staff *cannot* verify ORCID iDs on behalf of authors.

We look forward to receiving your revised manuscript.

Kind regards,

Alison Parker

Academic Editor

PLOS One

Journal Requirements:

When submitting your revision, we need you to address these additional requirements.

1. Please ensure that your manuscript meets PLOS ONE’s style requirements, including those for file naming. The PLOS ONE style templates can be found at

https://journals.plos.org/plosone/s/file?id=wjVg/PLOSOne_formatting_sample_main_body.pdf and

https://journals.plos.org/plosone/s/file?id=ba62/PLOSOne_formatting_sample_title_authors_affiliations.pdf.

2. We note that your Data Availability Statement is currently as follows: [Data is available in the manuscript. ]

Please confirm at this time whether or not your submission contains all raw data required to replicate the results of your study. Authors must share the “minimal data set” for their submission. PLOS defines the minimal data set to consist of the data required to replicate all study findings reported in the article, as well as related metadata and methods (https://journals.plos.org/plosone/s/data-availability#loc-minimal-data-set-definition).

For example, authors should submit the following data:

- The values behind the means, standard deviations and other measures reported;

- The values used to build graphs;

- The points extracted from images for analysis.

Authors do not need to submit their entire data set if only a portion of the data was used in the reported study.

If your submission does not contain these data, please either upload them as Supporting Information files or deposit them to a stable, public repository and provide us with the relevant URLs, DOIs, or accession numbers. For a list of recommended repositories, please see https://journals.plos.org/plosone/s/recommended-repositories.

If there are ethical or legal restrictions on sharing a de-identified data set, please explain them in detail (e.g., data contain potentially sensitive information, data are owned by a third-party organization, etc.) and who has imposed them (e.g., an ethics committee). Please also provide contact information for a data access committee, ethics committee, or other institutional body to which data requests may be sent. If data are owned by a third party, please indicate how others may request data access.

3. Please include captions for your Supporting Information files at the end of your manuscript, and update any in-text citations to match accordingly. Please see our Supporting Information guidelines for more information: http://journals.plos.org/plosone/s/supporting-information.

4. If the reviewer comments include a recommendation to cite specific previously published works, please review and evaluate these publications to determine whether they are relevant and should be cited. There is no requirement to cite these works unless the editor has indicated otherwise.

5. Please review your reference list to ensure that it is complete and correct. If you have cited papers that have been retracted, please include the rationale for doing so in the manuscript text, or remove these references and replace them with relevant current references. Any changes to the reference list should be mentioned in the rebuttal letter that accompanies your revised manuscript. If you need to cite a retracted article, indicate the article’s retracted status in the References list and also include a citation and full reference for the retraction notice.

[Note: HTML markup is below. Please do not edit.]

Reviewers' comments:

Reviewer’s Responses to Questions

**Comments to the Author**

1. Is the manuscript technically sound, and do the data support the conclusions?

Reviewer #1: Yes

Reviewer #2: Yes

**********

2. Has the statistical analysis been performed appropriately and rigorously?

Reviewer #1: N/A

Reviewer #2: Yes

**********

3. Have the authors made all data underlying the findings in their manuscript fully available?

The PLOS Data policy

Reviewer #1: Yes

Reviewer #2: Yes

**********

4. Is the manuscript presented in an intelligible fashion and written in standard English?

Reviewer #1: Yes

Reviewer #2: Yes

**********

Reviewer #1: A very interesting review of a relatively neglected area of discussion at a wider level. I think the paper is well written, structured well and the review has followed a rigourous process. I have some reflections which would help to strengthen the paper further.

In the introduction, it may be worth adding some detail about the policy context for MHH in India e.g. SBM and the various national schemes in place to set the tone, and to help to position the paper’s focus on tribal communities. There is reference to tribal communities as making up 8.6% of India’s population- Given that tribal communities are such a small percentage and in isolated areas, can you please expand a little more on the details about the cultural practices, poverty and lack of healthcare services and the impact this has for them?

The paper makes reference to government schemes for tribal populations at the top of page 10. lt may be worth clarifying which government schemes have been put in place already, or at least to give some examples in relation to tribal communities

I appreciate the strength of the well-designed search strategy. Did the search also specify "India" as a term?

An overall bit of feedback for the results section: as this is a review, I would reference each publication throughout the whole section. What was classed as 'media' as part of the review- was social media included? It may be worth specifying this more clearly. In the section on school absenteeism, a reference is needed for the 14.7% statistic on teachers.

I would urge caution about saying MHM is the critical issue for public health in India, and maybe loosen the language at the start of the discussion section.

Throughout the text, it might be worth adding in the geographies/states the studies are focused on, to deepen the understanding of the issues in different contexts in India. This would make it more relatable and easier to understand the findings

I would also recommend you clarify the language a little throughout the discussion - repeated references to 'studies' is confusing, and I would advise changing to "[name] et al found that...."

Overall, this is a thorough and important review on a niche and neglected area in MHH in India.

Reviewer #2: The manuscript is technically sound, employing a well-structured and rigorous systematic review methodology. A commendable aspect of this work is the complete availability and transparency of the underlying data throughout the manuscript and supplementary material, which ensures the full reproducibility of the study. The collected evidence logically and consistently substantiates the presented analyses and conclusions. By addressing the specific socioeconomic and cultural nuances pertaining to tribal adolescent girls in India, this study addresses a critical gap in the existing literature on menstrual health and dignity. Given its scientific rigor and robust empirical foundation, I consider this manuscript to make a valuable contribution to the field.

**********

what does this mean?). If published, this will include your full peer review and any attached files.

If you choose “no”, your identity will remain anonymous but your review may still be made public.

**Do you want your identity to be public for this peer review?** For information about this choice, including consent withdrawal, please see our Privacy Policy

Reviewer #1: **Yes:**  Amita Bhakta

Reviewer #2: **Yes:**  Amanda Guimaraes Cunha

**********

[NOTE: If reviewer comments were submitted as an attachment file, they will be attached to this email and accessible via the submission site. Please log into your account, locate the manuscript record, and check for the action link "View Attachments". If this link does not appear, there are no attachment files.]

To ensure your figures meet our technical requirements, please review our figure guidelines: https://journals.plos.org/plosone/s/figures

You may also use PLOS’s free figure tool, NAAS, to help you prepare publication quality figures: https://journals.plos.org/plosone/s/figures#loc-tools-for-figure-preparation.

NAAS will assess whether your figures meet our technical requirements by comparing each figure against our figure specifications.

# Author comment: Author response to Decision Letter 1

**DOI:** 10.1371/journal.pone.0360401.r002

10 Sep 2026

Reviewer #1: A very interesting review of a relatively neglected area of discussion at a wider level. I think the paper is well written, well structured, and the review has followed a rigorous process. I have some reflections which would help to strengthen the paper further.

1. In the introduction, it may be worth adding some detail about the policy context for MHH in India, e.g. SBM and the various national schemes in place to set the tone, and to help to position the paper’s focus on tribal communities. There is reference to tribal communities as making up 8.6% of India’s population. Given that tribal communities are such a small percentage and in isolated areas, can you please expand a little more on the details about the cultural practices, poverty and lack of healthcare services and the impact this has on them?

Answer: Thank you for this valuable suggestion. We have updated the we have expanded the Introduction to provide greater context on India’s policy landscape for menstrual health and hygiene (including SBM and relevant national schemes), and to elaborate on the cultural practices, poverty, limited healthcare access, and related challenges affecting tribal communities.

2. The paper refers to government schemes for tribal populations at the top of page 10. It may be worth clarifying which government schemes have been put in place already, or at least to give some examples in relation to tribal communities.

Answer: Thank you for this important remark. We have rephrased the paragraph and added similar government schemes.

3. I appreciate the strength of the well-designed search strategy. Did the search also specify "India" as a term?

Answer: Yes, we have mentioned “India” as a term in the search strategy and updated in the search terms used in the manuscript.

4. An overall bit of feedback for the results section: as this is a review, I would reference each publication throughout the whole section. What was classed as 'media' as part of the review- was social media included? It may be worth specifying this more clearly. In the section on school absenteeism, a reference is needed for the 14.7% statistic on teachers.

Answer: Thank you for this helpful feedback; we have revised the Results section to consistently cite the included publications, clarified that “media” included social media where applicable, and added the appropriate reference for the reported 14.7% statistic on teachers.

5. I would urge caution about saying MHM is the critical issue for public health in India, and maybe loosen the language at the start of the discussion section.

Answer: Thank you for this important observation. We agree that describing menstrual health management (MHM) as “the critical issue” in India may overstate the evidence presented in our review. We have therefore revised the opening of the Discussion to use more cautious and evidence-based language. Specifically, the phrase “The critical issue in India’s public health” has been replaced with “Menstrual health management (MHM) remains an important public health and social concern, particularly among adolescent girls and women from marginalised communities in India.” This revision better reflects the scope and findings of our review while avoiding an overly broad national claim.

6. Throughout the text, it might be worth adding the geographies/states the studies are focused on, to deepen the understanding of the issues in different contexts in India. This would make it more relatable and easier to understand the findings.

Answer: We have updated as per the suggestion in the results and discussion section. State and geographical location.

7. I would also recommend you clarify the language a little throughout the discussion - repeated references to 'studies' is confusing, and I would advise changing to "[name] et al found that...."

Answer: We have updated the changes as per suggested.

Overall, this is a thorough and important review on a niche and neglected area in MHH in India.

Attachment Submitted filename: Reviewer comment plos.docx

# Peer review: Decision Letter 1

**DOI:** 10.1371/journal.pone.0360401.r003

**Authors:** Alison Parker

27 Sep 2026

Menstrual health challenges and associated barriers among tribal adolescent girls in India: A systematic review

PONE-D-26-27585R1

Dear Dr. Begum,

We’re pleased to inform you that your manuscript has been judged scientifically suitable for publication and will be formally accepted for publication once it meets all outstanding technical requirements.

Within one week, you’ll receive an e-mail detailing the required amendments. When these have been addressed, you’ll receive a formal acceptance letter and your manuscript will be scheduled for publication.

An invoice will be generated when your article is formally accepted. Please note, if your institution has a publishing partnership with PLOS and your article meets the relevant criteria, all or part of your publication costs will be covered. Please make sure your user information is up-to-date by logging into Editorial Manager at Editorial Manager® and clicking the ‘Update My Information' link at the top of the page. For questions related to billing, please contact billing support.

If your institution or institutions have a press office, please notify them about your upcoming paper to help maximize its impact. If they’ll be preparing press materials, please inform our press team as soon as possible -- no later than 48 hours after receiving the formal acceptance. Your manuscript will remain under strict press embargo until 2 pm Eastern Time on the date of publication. For more information, please contact onepress@plos.org.

Kind regards,

Alison Parker

Academic Editor

PLOS One

Additional Editor Comments (optional):

Reviewers' comments:

Reviewer’s Responses to Questions

**Comments to the Author**

Reviewer #1: All comments have been addressed

**********

2. Is the manuscript technically sound, and do the data support the conclusions?

Reviewer #1: Yes

**********

3. Has the statistical analysis been performed appropriately and rigorously?

Reviewer #1: N/A

**********

4. Have the authors made all data underlying the findings in their manuscript fully available?

The PLOS Data policy

Reviewer #1: Yes

**********

5. Is the manuscript presented in an intelligible fashion and written in standard English?

Reviewer #1: Yes

**********

Reviewer #1: Thank you for addressing the comments, I am satisfied that my feedback has been well addressed on this important paper.

**********

what does this mean?). If published, this will include your full peer review and any attached files.

If you choose “no”, your identity will remain anonymous but your review may still be made public.

**Do you want your identity to be public for this peer review?** For information about this choice, including consent withdrawal, please see our Privacy Policy

Reviewer #1: **Yes:**  Amita Bhakta

**********

# Editor report: Acceptance letter

**DOI:** 10.1371/journal.pone.0360401.r004

**Authors:** Alison Parker

PONE-D-26-27585R1

PLOS One

Dear Dr. Begum,

I'm pleased to inform you that your manuscript has been deemed suitable for publication in PLOS One. Congratulations! Your manuscript is now being handed over to our production team.

At this stage, our production department will prepare your paper for publication. This includes ensuring the following:

* All references, tables, and figures are properly cited

* All relevant supporting information is included in the manuscript submission,

* There are no issues that prevent the paper from being properly typeset

You will receive further instructions from the production team, including instructions on how to review your proof when it is ready. Please keep in mind that we are working through a large volume of accepted articles, so please give us a few days to review your paper and let you know the next and final steps.

Lastly, if your institution or institutions have a press office, please let them know about your upcoming paper now to help maximize its impact. If they'll be preparing press materials, please inform our press team within the next 48 hours. Your manuscript will remain under strict press embargo until 2 pm Eastern Time on the date of publication. For more information, please contact onepress@plos.org.

You will receive an invoice from PLOS for your publication fee after your manuscript has reached the completed accept phase. If you receive an email requesting payment before acceptance or for any other service, this may be a phishing scheme. Learn how to identify phishing emails and protect your accounts at https://explore.plos.org/phishing.

If we can help with anything else, please email us at customercare@plos.org.

Thank you for submitting your work to PLOS One and supporting open access.

Kind regards,

PLOS One Editorial Office Staff

on behalf of

Dr. Alison Parker

Academic Editor

PLOS One
